# Proprietary Preparations

**Published:** 1908-01-18

**Authors:** 


### The Hospital

A Journal of

The Medical Sciences and Hospital Administration.

New Series. No. 44, Vol. II. [No. 1116, Vol. XLIII.] SATURDAY, JANUARY 18, 1908.

### PROPRIETARY PREPARATIONS.

For various reasons considerable attention has recently been directed to the numerous medicinal preparations which are put on the market by individual pharmacists or by firms of manufacturing druggists. It cannot be doubted that the number of these preparations is an increasing one, and it is equally certain that they are to a greater and greater extent brought before the attention of the profession, and sometimes, also, of the public, by the aid of advertisement. As a consequence, it is suggested, certain evils have come into existence, and further, that steps ought to be taken to neutralise these evils. First of all comes the assertion that numerous private formulæ are pressed so strongly on the attention of the profession that prescribing in the old meaning of the term has become a lost art. The young practitioner, it is alleged, finds his prescriptions ready-made, and consequently neglects the official remedies set forth in the pages of the *British Pharmacopœia*. Following on this, so the accusation runs, there arises prejudice to the art and craft of pharmacy. The dispenser, instead of utilising his skill and knowledge in the preparation of the extemporaneous prescriptions of the physician, is reduced to the position of a mere retailer of ready-made goods. And last, but by no means least, it is urged that the public finds it easy to purchase preparations which enjoy a quasi-medical sanction, and so the practices of drug-taking and of amateur prescribing are encouraged. As a remedy for all these habits, members of the profession are being advised to avoid the use of proprietary preparations, and to cultivate the writing of prescriptions in which medicines owning no individual commercial relationship are associated and combined. It seems worth while briefly to examine these several positions, in order to see how far they correspond with actual facts, and to what extent it is possible or desirable to attempt to modify them.

In the first place it must, we think, be admitted that there is at least a measure of truth in the assertion that the art of prescribing has somewhat declined. But we doubt whether the entire blame for this is to be cast on the manufacturing druggist. The crowded condition of the medical curriculum, and the associated neglect of practical pharmacy by the medical teacher and the medical student, must take some share of the responsibility. Again, it is necessary to remember that the administration of drugs has a much less prominent place in the scheme of medical treatment than was formerly the case. In the days of an abounding polypharmacy the art of prescribing was an essential qualification for practice; but as prescriptions have become, perhaps, fewer in number, and certainly more simple in structure, the range of necessity in this direction has become much more restricted. Hence, personally, we are hardly prepared to recognise that skill in the construction of prescriptions on traditional lines is so urgent and essential a claim as some authorities hold it to be. At the same time we have no hesitation in saying that the physician should be as familiar with the chemical and physical qualities of the drugs he employs as he is with their pharmacological actions and their therapeutic values. In so far as this is not the case, medical training needs to be amended, and if there has been deficiency in this respect before graduation, that is all the more reason why later opportunities should be developed. Now arises the question how far the principle just defined applies to the use of proprietary medicinal preparations. It is, we think, fairly manifest that the more complete the knowledge which the practitioner possesses of drugs and other medicines of well-established reputation, the less necessity will he have for the products of private commercial enterprise. The art of using drugs is not of yesterday, and no one can doubt that for most of the purposes for which drugs are given, well-known remedies are already in existence, and enjoy authoritative recognition. At the same time, both medicine and pharmacy are progressive arts, and improvements and advances which have a commercial motive may still be worthy of professional attention. Hence we think that the physician is right to maintain his freedom to prescribe any drug, whether new or old, which he has reason to believe will help his patients. And we quite allow that a private pharmacist or manufacturing firm who introduces or develops some new medicinal method may reasonably conduct the business which ensues on a strictly financial basis. Although a physician, as a member of a profession, cannot profit by a transaction of this order, there is not the same claim on those who admittedly are engaged in commercial pursuits. From all this it follows that there can be no restriction on the development of new remedies and new combinations, and no moral demand that the physician shall not employ them, assuming, that is, that their exact nature and composition are declared. Only, as already intimated, the practitioner will find the less need for the use of new remedies in proportion as he is familiar with the older ones, and he will remember that there are interests other than the welfare of the patient which inspire the manufacturers of proprietary preparations.

As regards the pharmacists and the public these must, it seems to us, accept the consequences of the position above defined. If the physician is convinced of the value of a new remedy it is his duty to prescribe it whether this is or is not convenient to the dispensers, and in spite of the fact that some members of the public may, as a result, use it on their own initiative and responsibility. We quite appreciate the efforts which are made by many pharmacists to prevent mere humbug and pretence from securing the countenance of members of the medical profession, and perseverance on these lines will probably bring an appropriate reward. But it must rest with the physician to select his own remedies, and when the choice is made the dispenser is bound to adjust himself to the demand.

